# Study of Liposomes Containing Extract from the Leaves of *Protium heptaphyllum* (Aubl.) March in Animals Submitted to a Mutagenic Model Induced by Cyclophosphamide

**DOI:** 10.3390/biology13090706

**Published:** 2024-09-08

**Authors:** Naiéle Sartori Patias, Valéria Dornelles Gindri Sinhorin, Ana Júlia Lopes Braga Ferneda, João Maurício Andrade Ferneda, Marina Mariko Sugui, Stela Regina Ferrarini, Gisele Facholi Bomfim, Joaz Wellington Lopes, Nadia Aline Bobbi Antoniassi, Larissa Cavalheiro, Nelson Luís de Campos Domingues, Adilson Paulo Sinhorin

**Affiliations:** 1 Postgraduate Program in Biotechnology and Biodiversity of the Pro Centro-Oeste Network, Federal University of Mato Grosso, Sinop 78550-728, Brazil; nai.sartori@gmail.com (N.S.P.); nelsondomingues@ufgd.edu.br (N.L.d.C.D.); 2 Department of Chemistry, Institute of Exact and Earth Sciences, Federal University of Mato Grosso, Cuiabá 78060-719, Brazil; 3 Postgraduate Program in Health Sciences, Federal University of Mato Grosso, Sinop 78550-728, Brazil; ajlbraga@hotmail.com (A.J.L.B.F.); stela.ferrarini@ufmt.br (S.R.F.); gisele.bomfim@ufmt.br (G.F.B.); 4 Institute of Health Sciences, Federal University of Mato Grosso, Sinop 78550-728, Brazil; joaoferneda@hotmail.com (J.M.A.F.); marina.sugui@ufmt.br (M.M.S.); joazw.sinop@gmail.com (J.W.L.); nadia.antoniassi@ufmt.br (N.A.B.A.); 5 Animal Pathology Laboratory, Federal University of Mato Grosso, Sinop 78550-728, Brazil; 6 Institute of Natural, Human and Social Sciences, Federal University of Mato Grosso, Sinop 78550-728, Brazil

**Keywords:** oxidative stress, antimutagenic, cancer, flavonoid, quercetin

## Abstract

**Simple Summary:**

In this study, the protective effect of *Protium heptaphyllum* (*P. heptaphyllum*), a plant with a high flavonoid content, was analyzed against damage caused by cyclophosphamide (CPA), a chemotherapy drug known for its adverse effects. Using liposomes to transport the plant extract, an experiment was carried out with male Swiss mice that received the extract for 14 days before CPA. The results demonstrated that *P. heptaphyllum* liposomes reduced DNA damage and oxidative stress, evidenced by the increase in antioxidant enzymes such as superoxide dismutase (SOD) and glutathione peroxidase (GPx). There were also improvements in liver and kidney indicators, suggesting organ protection. In summary, these results highlight the potential of *P. heptaphyllum* liposomes as a natural chemical protection agent that can help reduce the side effects of cancer treatment, while providing antioxidant benefits.

**Abstract:**

Cyclophosphamide (CPA) is an alkylating agent used as a chemotherapy agent in the treatment of cancer, but it has immunosuppressive effects. *Protium heptaphyllum* (*P. heptaphyllum*) is a plant rich in triterpenes and flavonoids, with some bioactive and therapeutic properties presented in the literature. Thus, the present study aimed to investigate the chemoprotective potential of *P. heptaphyllum* extract inserted into liposomes against oxidative damage chemically induced by CPA. Male Swiss mice received 1.5 mg/kg of *P. heptaphyllum* liposomes as a pre-treatment for 14 consecutive days (via gavage) and 100 mg/kg of CPA in a single dose (via intraperitoneal) on the 15th day. After the experimental period, blood and organ samples were collected for histopathological and biochemical analyses, including superoxide dismutase (SOD), glutathione peroxidase (GPx), catalase (CAT), glutathione S-transferase (GST), reduced glutathione (GSH), thiobarbituric acid reactive substances (TBARS), ascorbic acid (ASA), carbonyl protein, cytokine measurement, and micronucleus testing. The results showed that liposomes containing *P. heptaphyllum* extract have an antimutagenic effect against damage induced to DNA by CPA, and that they also protect against oxidative stress, as verified by the increase in the antioxidant enzymes SOD and GPx. The improvement in alkaline phosphatase and creatinine markers suggests a beneficial effect on the liver and kidneys, respectively. However, the depletion of GSH in the liver and brain suggests the use of antioxidants for the metabolism of molecules generated in these tissues. In general, these data show good prospects for the use of *P. heptaphyllum* liposomes as a cancer chemoprotective agent, as well as possible antioxidant action, conceivably attributed to the flavonoids present in the plant extract.

## 1. Introduction

Cyclophosphamide (CPA) is a drug extensively used as an alkylating agent in the treatment of several malignant neoplasms, including breast cancer, multiple myeloma, kidney disease, rheumatoid arthritis, juvenile dermatomyositis, systemic sclerosis, interstitial lung disease, lupus vasculopathy, systemic vasculitis, and refractory treatment of thrombocytopenic purpura [1,2]. CPA can be administered orally or intravenously, with oral dosing administered daily (every 24 h) and in pulses, adjusting the dose according to hematologic and renal toxicity. Adverse reactions to CPA include bone marrow suppression, susceptibility to infections, sterility, amenorrhea, nephrotoxicity, and cystitis, as well as cardiovascular complications such as sinus bradycardia, pericarditis, myocarditis, and heart failure [1,2,3,4].

The search for new drugs that help treat pathologies while exerting fewer side effects is essential to improve patients’ quality of life and to advance medicine. The wealth of substances derived from plants has aroused great interest in this field among scientific community as a promising source for the development of new drugs. The search for innovative medicinal agents, especially those derived from natural sources, is driven by the presence of several molecules in the secondary metabolism of plants [5,6,7]. Many drugs originate directly or indirectly in plant compounds, and plants harbor a variety of phytoconstituents, each with unique and distinct properties [8]. 

*Protium heptaphyllum* (*P. heptaphyllum*) is a plant commonly known as almacega, almíscar or breu branco, and it is typically found in the Amazon [9]. Its leaves and resin are used in folk medicine due to their stimulating, anti-inflammatory, and healing properties [10,11]. It derives from the Burceraceae family, comprising 18 genera and more than 700 species divided into three tribes, with the genus Protium (tribe Protieae) being the main member of the family [12,13].

The resins, oils, and leaves of the genus Protium are extremely rich in terpenes and polyphenols, such as flavonoids [5,12,14]. Phenolic compounds, including flavonoids, are known as free radical scavengers and are highly efficient in preventing autoxidation. Studies indicate that flavonoids can reduce cellular stress, which includes neuroinflammation, oxidative stress, proteotoxicity, and endoplasmic reticulum stress [15]. Antioxidants, in addition to providing several beneficial effects, can inhibit or delay the emergence of tumor cells, delay aging, and prevent other cellular damage resulting from redox imbalance [16,17,18].

Liposome nanotechnology is one of the new, innovative therapeutic approaches based on plants. This approach improves the solubility, stability, and specific targeting of active substances, overcoming absorption and bioavailability challenges [19]. By encapsulating an active substance, liposomes offer controlled release, minimize side effects, and maximize therapeutic efficacy, presenting a promising frontier for the optimization of phytotherapeutic treatments in contemporary medicine [19,20]. Thus, herbal medicines conjugated using new technologies, such as using liposomes, can aid in treatment by offering effective, accessible solutions, with fewer side effects, for various pathologies due to the variety of chemical compounds, such as saponins, flavonoids, and catechins, present in plant species, highlighting the need for studies on the biological activity and toxicity of these plants to encourage investments from the pharmaceutical industry [16,17,21]. Therefore, this research sought to investigate the preventive chemoprotective effects of the ethyl acetate fraction of *P. heptaphyllum* extract inserted in liposomes against the oxidative processes caused by CPA.

## 2. Material and Methods

### 2.1. Preparation of Extract and Development of Liposomes

The work was developed at the Integrated Chemical Sciences Research Laboratories (LIPEQ), located at the Federal University of Mato Grosso, Campus of Sinop. The exsiccata is registered in the collection of the Centro-Norte Mato-Grossense Herbarium (CNMT) of the Federal University of Mato Grosso, located at the Campus of Sinop, under number 625.

The leaves of the species *P. heptaphyllum* were collected and selected, dried, crushed into powder, and subsequently macerated with ethanol for 7 days. Next, 1.05223 kg of leaf powder were mixed with 4 L of ethanol. After the maceration period, chlorophyll was removed using activated charcoal (69.57 g). Then, the extract underwent the solvent rotary evaporation process, followed by lyophilization to obtain the crude ethanolic extract (CEE), and was finally subjected to functional group identification tests. A portion of the CEE was fractionated through a silica gel chromatographic column, using a solvent gradient of increasing polarity; additional details can be found in the work of Patias et al. [14]. After fractionation, the choice was made to employ the ethyl acetate fraction (EAF) of *P. heptaphyllum* in the development of liposomes (LP) due to its high concentration of flavonoids.

The liposomes were developed using the reverse-phase evaporation method, followed by lipid film extrusion, as described by Hua and Wu [22].

The liposomes were characterized physicochemically by evaluating the active compound content and average micelle diameter using a Mastersizer^®^ 2000 device (Malvern, England); the polydispersity index (PDI) using photon brightness spectroscopy analysis (Zetasizer^®^ NanoZS model ZEN 3600, Malvern Instruments, (Malvern, England); the zeta potential by electrophoretic mobility; pH in a potentiometer (Denver^®^ Instrument VB-10, New York, NY, USA); as well as the dosage of quercetin in the nanosystem. The results obtained from the characterization of *P. heptaphyllum* liposomes show that the technique used allowed the formation of particles on the nanometric scale. Micelles with small size (232 ± 1.70) and low polydispersity index (0.21 ± 0.01) characterize a system with low size distribution. The zeta potential value demonstrates the surface charge potential (−20.07 ± 1.10), which is a fundamental parameter to predict the stability of liposomes. Zeta potential values close to −20 mV (as occurs with the *P. heptaphyllum* liposomes in the present study) can be considered satisfactory to maintain the stability of the system [18]. The charges found in the developed liposomal formulations follow the standard for formulations based on DPPC, in which the occurrence of a negative charge is predominant among the samples.

### 2.2. In Vivo Tests

Initially, the Malone hippocratic test was performed to construct a dose curve for liposomes containing *P. heptaphyllum* extract at concentrations of 0.5, 1.0, 1.5, 2.5, and 5 mg/kg, using three animals for each concentration. This procedure aimed to analyze the potential acute toxicity of the doses used, as described by Malone [23]. Based on the results obtained, a specific dose of 1.5 mg/kg was then chosen to be administered to the animals.

#### 2.2.1. Animals and Experimental Design

Male Swiss mice weighing an average of 35 g ± 3 g were used for the study. All steps for authorization to work with animals were approved by the Guidelines of the Ethics Committee for the Use of Animals of the Federal University of Mato Grosso, No. 23108.030996/2022-07. A total of eight animals per group were employed for a treatment period of 14 days.

Throughout the experimental period, the animals were kept in polypropylene boxes with a controlled temperature of 22 ± 2 °C and a 12 h light–dark cycle, with free access to water and food. The animals were distributed into four experimental groups according to whether or not they were treated with liposomes containing *P. heptaphyllum* leaf extract, as follows:

Group C: Control; CPA Group: cyclophosphamide; LP Group: liposome with *P. heptaphyllum* extract; LP + CPA Group: liposome with *P. heptaphyllum* extract + cyclophosphamide.

The acclimatization period was 2 weeks. The groups treated with liposomes containing the EAF extract (LP, LP + CPA) received a dose of 1.5 mg/kg orally (v.o.), once a day, for 14 days. The Control groups (C and CPA) received water solution (H_2_O) in the same volume, v.o., also for 14 days.

CPA was supplied by Baxter^®^, diluted in 0.9% saline solution, and administered to the animals intraperitoneally (i.p.) on the 15th day at a concentration of 100 mg/kg (0.1 mL/10 g body weight) [24].

After the stipulated period, the animals were fasted for 8 h, and at the end of the 16th day, blood was collected by cardiac puncture under anesthesia (Chlortamine^®^ (Ketamine, 50 mg/kg) and Rompun^®^ (Xylazine, 20 mg/kg)); the animals were euthanized by cervical dislocation. Samples of the liver, kidneys, heart, and brain were collected by dissection, washed with isotonic saline solution, and weighed to determine absolute (g) and relative (g/100 g body weight) weight. In addition, the weight of the mice (g) was assessed; part of the tissue samples were stored frozen at −80 °C and part fixed in 10% phosphate-buffered formalin for subsequent analysis. Plasma was obtained from whole blood after centrifugation (4000 rpm, 10 min; Nova Técnica, NT-835, Piracicaba, Brazil).

#### 2.2.2. Analysis of Anthropometric Measurements

Body weight, as well as daily water and food consumption, were assessed at the beginning and end of the experiment. To analyze consumption, 500 g of food were placed in the box, and the uneaten food was weighed every two days. The difference represented the amount of food consumed by the mice in the box, expressed in grams. The same protocol was used to analyze water consumption, but in mL measurements, where 500 mL of water were offered. This analysis was performed throughout the treatment.

#### 2.2.3. Micronucleus Test

The preparation and collection of bone marrow erythrocyte slides for evaluation of micronucleus (MN) frequency followed the methodology proposed by MacGregor et al. [25]. For each animal, duplicate smears were created and stained to differentiate polychromatic erythrocytes (PCE) from normochromic erythrocytes (NCE). From these prepared slides, 1000 cells per animal were analyzed under a light microscope (Kasvi/Olen, K55-BA, Pinhais, PR, Brazil), with 1000× magnification (immersion). The material was analyzed in a blind test, and the slides were decoded at the end of the analyses.

#### 2.2.4. Biochemical Analyses of Liver, Kidney, Brain, Heart, and Plasma

The biochemical parameters (glucose, aspartate aminotransferase—AST, alkaline phosphatase—ALP, total cholesterol, triglycerides, and creatinine) of blood plasma were analyzed using commercial kits (Labtest^®^, Diagnóstico S. A., Minas Gerais, Brazil).

The enzymatic antioxidant activity of CAT (catalase) was determined according to the methods of Nelson and Kiesow [26], SOD (superoxide dismutase) according to the suggestions of Misra and Fridovich [27], GST (glutathione S-transferase) according to the work of Habig et al. [28], and GPx (glutathione peroxidase) was measured according to the techniques of Paglia and Valentine [29]. The dosage of the non-enzymatic antioxidants GSH (reduced glutathione) was evaluated according to the methods of Sedlack and Lindsay [30], and ascorbic acid (ASA) was measured according to the suggestions of Roe [31]. The indirect markers of oxidative damage evaluated included TBARS (thiobarbituric acid reactive substances) and carbonyl (carbonylated proteins), according to the technique described by Buege and Aust and Colombo et al., respectively [32,33]. The protein content of the tissues was determined according to the work of Bradford [34]. All analyses were performed using a UV–Vis spectrophotometer (Varian Cary 50 Scan, Northfield, MI, USA).

#### 2.2.5. Histological Analysis

Liver and kidney fragments were used for histopathological analysis. These were fixed in 10% phosphate-buffered formalin for 48 h and immersed in 70% alcohol. The tissue fragments were cleaved to a thickness of 0.4 cm, placed in cassettes, and subjected to the dehydration and clearing process in an automatic histotechnician (Lupitec, PT 05 TS, São Carlos, SP, Brazil). They were later embedded in paraffin and cut to a thickness of 3 μm using a semi-automatic microtome (Leica Biosysthems RM2245, Nussloch, Germany). The slides were stained with hematoxylin and eosin and evaluated under an optical microscope (Nikon eclipse 80I, Tokyo, Japan). Histological evaluation was carried out along the entire length of the slide fragment, with magnification objectives of 10, 40, and 60 times. A trained technician evaluated the occurrence of circulatory, degenerative, proliferative, necrotic, and inflammatory changes. The changes were altered in terms of distribution and intensity, according to the methods of Barros et al. (2021) [35].

#### 2.2.6. Immunological Analysis by ELISA

To perform immunological analyses of cytokines, such as tumor necrosis factor alpha (TNF-α), interferon gamma (IFN-γ), interleukin 6 (IL-6), interleukin 10 (IL-10), interleukin 17 (IL-17), and interleukin 1β (IL-1β), the enzyme-linked immunosorbent assay (ELISA) methodology was employed using commercial kits from DIY Duoset Elisa^®^, according to the instructions provided by the manufacturer.

### 2.3. Data Analysis

The data were initially subjected to the Kolmogorov–Smirnov normality test. Then, a two-way analysis of variance (ANOVA) was performed, followed by the Tukey–Kramer multiple comparison test to assess the means when more than two groups were present. In cases where the results did not meet the normality criteria, the Kruskal–Wallis test was used, followed by Dunn’s post-test. The acceptable significance level adopted was *p* < 0.05. The results were presented as mean ± standard deviation or median and total range, according to the protection obtained. Graphs were created and statistical analysis was performed using Graph Pad Prism 8.0 statistical software.

The frequency of micronucleated polychromatic erythrocytes (MCPEs) in the different experimental groups was compared using the Chi-squared test [36]. The percentage of damage reduction (decrease in the mean frequency of micronucleated cells) of the extracts that showed antimutagenic activity were calculated according to the method of Waters et al. [37], using the following formula:

(1) $$\%\;reduction=\frac{Frequency\;of\;PCEMs\;in\;a-Frequency\;of\;PCEMs\;in\;b\;}{Frequency\;of\;PCEMs\;in\;a-Frequency\;of\;PCEMs\;in\;c}\times 100$$

where:

*a* = group treated with CPA;

*b* = group treated with LP + CPA;

*c* = group treated with 0.9% NaCl (control).

## 3. Results 

The measurements of initial body weight, final body weight, and feed and water consumption did not show statistically significant differences between the groups analyzed, as shown in Table 1. However, there were changes in the weights of the organs investigated, such as the liver and heart. In the liver, the weight increased in the CPA group when compared to that in the C group, and the LP + CPA group showed a reduction in weight when compared to that of the LP and CPA groups. For the heart, the CPA group showed an increase in weight compared to that of the C group, and in the LP + CPA group, there was a decrease when compared to the results for the CPA group, as indicated in Table 1. The other organs investigated did not show statistically significant changes.

Plasma glucose, cholesterol and aspartate aminotransferase (AST) enzyme activity levels did not show statistically significant changes in the groups analyzed. There was a decrease in alkaline phosphatase (ALP) activity in the LP + CPA group compared to that in group C. In addition, creatinine levels were reduced in the CPA, LP, and LP + CPA groups compared to those noted in group C. As for triglycerides, a decrease was observed in the CPA group compared to the levels in group C, as shown in Table 2.

Table 3 shows the frequency of micronucleated polychromatic erythrocytes (PCEMN) after the pretreatment of mice with liposomes containing *P. heptaphyllum* extract on chemically induced damage by CPA. The group treated with LP + CPA showed a significant reduction (*p* ≤ 0.05) in the frequency of micronuclei when compared with that of the positive control group, showing a chemoprotective effect of the extract and thus, the possibility of producing benefits related to the prevention of DNA damage as an antimutagenic agent. On the other hand, the group treated only with the liposome containing the extract did not show a mutagenic effect when compared with the negative control group.

We did not observe statistical changes between the groups studied for cytokine levels in the liver and kidney tissue (Table 4).

**Figure 1:**
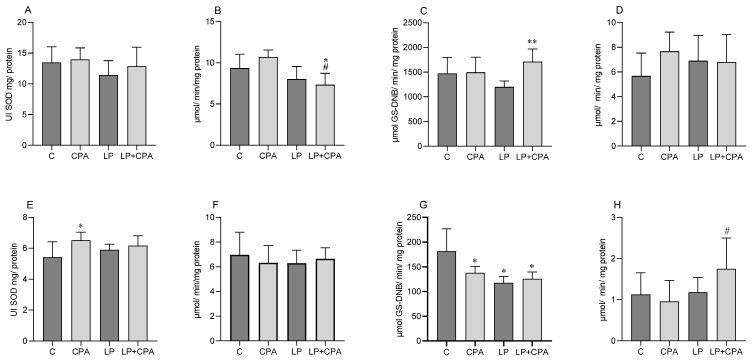
Analysis of enzymatic activities of male Swiss mice treated with LP (protium liposome) and CPA (cyclophosphamide). SOD: superoxide dismutase (**A**), CAT: catalase (**B**), GST: glutathione S-transferase (**C**), e GP_X_: glutathione peroxidase (**D**) in the liver; SOD: superoxide dismutase (**E**), CAT: catalase (**F**), GST: glutathione S-transferase (**G**), e GPx: glutathione peroxidase (**H**) in the kidney. The results are presented as mean ± standard deviation. ANOVA (two-way), followed by Tukey’s post-hoc test. * *p* < 0.05 vs. C; ** *p* < 0.05 vs. LP; # *p* < 0.05 vs. CPA; (n = 8).

In the liver, a decrease in the activity of the catalase enzyme was observed in the LP + CPA group compared to that in the C groups and the CPA group (Figure 1B). On the other hand, the GST enzyme showed an increase in activity in the LP + CPA group compared to the LP group (Figure 1C). As for the SOD and GPx enzymes, no statistically significant changes were observed (Figure 1A and Figure 1D, respectively). In the renal tissue, an increase in the activity of the SOD enzyme was observed in the CPA group compared to that in the C group (Figure 1E). No significant differences were observed in the activity of the catalase enzyme between the groups studied (Figure 1F), while the GST enzyme showed decreased activity in all treated groups, as illustrated in Figure 1G. The GPx enzyme increased its activity in the LP + CPA group when compared to that in the CPA group (Figure 1H).

GSH levels in the liver tissue decreased in the CPA, LP, and LP + CPA groups compared to those found in group C. In addition, carbonyl protein levels (carbonyl) increased in the CPA group compared to those in group C (Table 5). The other parameters analyzed in the liver tissue (ASA and TBARS) did not show significant changes in their results when compared to those of the control group (C) (Table 5). No statistical differences were observed in redox state markers such as GSH, ASA, and carbonyl in the renal tissue of the animals analyzed. TBARS showed a significant difference, with a decrease in the LP group compared to the levels in group C (Table 4).

We did not observe any significant changes in the histological analyses. 

**Figure 2:**
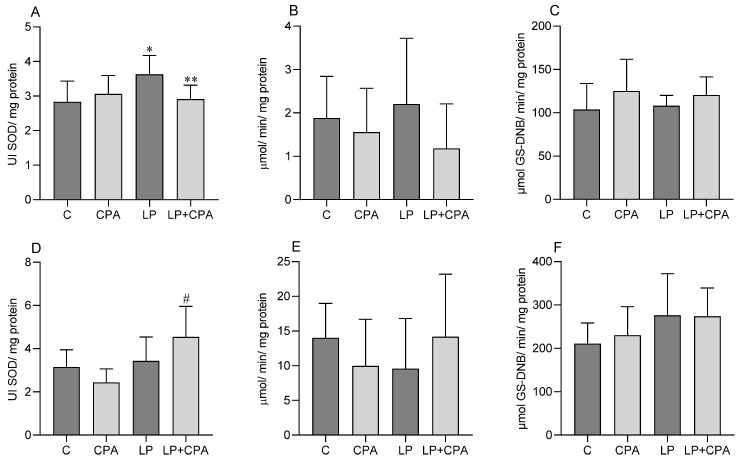
Analysis of enzymatic activities of male Swiss mice treated with LP (protium liposome) and CPA (cyclophosphamide). SOD: superoxide dismutase (**A**), CAT: catalase (**B**), GST: glutathione S-transferase (**C**), e GP_X_: glutathione peroxidase (**D**) in the brain; SOD: superoxide dismutase (**E**), CAT: catalase (**F**), GST: glutathione S-transferase. The results are presented as mean ± standard deviation. ANOVA (two-way), followed by Tukey’s post-hoc test. For analysis of GST (**B**) in brain tissue and CAT (**E**) in cardiac tissue, the Kruskal–Wallis test, followed by the post-hoc Dunn test, was used, and values were expressed as median and total range. * *p* < 0.05 vs. C; ** *p* < 0.05 vs. LP; # *p* < 0.05 vs. CPA; (n = 8).

The activity of the SOD enzyme in brain tissue increased in the LP group compared to that observed in the control group. However, in the LP + CPA group, a reduction in this activity was observed in relation to the LP group (Figure 2A). Regarding the activity of the CAT and GST enzymes, no statistically significant changes were detected (Figure 2B,C).

In cardiac tissue, a significant increase in the activity of the SOD enzyme was observed in the group treated with LP + CPA compared to the group that received only CPA (Figure 2D). Regarding the analysis of the activities of catalase and GST, no changes were identified between the groups analyzed (Figure 2E,F).

We observed a reduction in GSH levels in the brain tissue in the LP + CPA groups compared to the levels in the CPA group. Regarding the levels of ASA, TBARS, and carbonyl, no statistically significant differences were identified between the groups investigated (Table 6).

Regarding the markers GSH, TBARS, and carbonyl, in the cardiac tissue, no statistically significant changes were identified between the groups investigated. However, regarding the dosage of ASA, a decrease in its levels was observed in the group treated with CPA compared to those in the Control group (Table 6).

## 4. Discussion

It is known that polyphenols, especially flavonoids, exhibit several pharmacological attributes, such as antioxidant, anti-inflammatory, and anticancer properties, but their usage is still minor compared to their immense therapeutic potential [38]. Although many investigations have been carried out by several authors to study the protective effects of polyphenols, especially flavonoids, obtained from different plant extracts against various types of chemical residues in animal experiments, it is suggested that flavonoids are directly effective for antioxidant activities via the neutralization of free radicals [39]. Many extracts exhibit low bioavailability and rapid degradation, which limits their clinical efficacy. Encapsulating them in liposomes can significantly improve their stability, bioavailability, and specific targeting to tissues [38]. This synergy between phytotherapy and nanosystems allows for the creation of more effective treatments, with less toxicity and fewer side effects, marking a promising advancement in the area [38,40]. In this work, we sought to improve the use of the ethyl acetate fraction from the crude extract of *P. heptaphyllum* in the form of liposomes, and the results obtained revealed several interesting aspects related to the impact of liposomes containing the plant extract during 14 days of preventive treatment against mutagenesis subsequently induced for 24 h with CPA.

The results showed no statistically significant difference in initial or final body weight, as well as feed and water consumption, between the groups analyzed here, and similar data were found in the study by Patias et al. [18], where Wistar rats were treated with the same plant formulation, but at a dose of 1 mg/mL. In a study using mice over a period of 21 days, treatment with CPA (100 mg/kg) and metformin (3 mg/mL) significantly decreased body weight, in addition to reducing the survival rate of the animals [41]. On the other hand, it was observed that the flavonoid quercetin has a significant protective capacity against changes induced by CPA (150 mg/kg) in rats, reversing the decrease in body weight and food intake [42], which suggests that in our study, LP, administered for 14 days, and CPA for 24 h, were not toxic to the point of altering these parameters.

Among our findings regarding organ weight, an important observation was the statistical increase in liver and heart weight in the CPA group compared to the levels in the control group (C). This increase in weight could suggest that CPA may have a direct impact on the morphology and function of these organs. When we refer to liver weight, this may be linked to a possible hepatotoxic effect of CPA, since the liver is often the target of toxicity caused by chemotherapeutic agents or may even be indicative of inflammation or liver damage, often associated with CPA treatment [43]. Another observation was that pretreatment with LP in animals exposed to CPA (LP + CPA) caused a reduction in liver and heart weights compared to those observed in the groups that received only CPA. This finding could indicate that LP may have the ability to reduce the adverse effects caused by CPA in these organs, even if they were only exposed to the mutagenic agent for 24 h. Similarly, Dolgava et al. demonstrated that CPA treatment can affect heart and liver weight in mice [44].

Plasma analysis showed no change in AST in the treated groups, but indicated a decrease in ALP activity, a hepatobiliary marker, in the LP + CPA group, and in creatinine levels, a marker of renal function, for all groups treated with LP and CPA. In contrast, plasma triglyceride levels were elevated in the group treated with CPA alone, possibly due to changes in lipid metabolism caused by CPA. Studies by our group using *P. heptaphyllum* liposomes in the treatment of obesity caused by a high-calorie diet in Wistar rats showed a reduction in AST activity and creatinine levels in both the groups with liposome-containing extract and in the obese group. A similar reduction in ALP was also noted in the obese group and in the group treated with liposomes [18]. On the other hand, in the study by Ref. [14], the administration of EAF from this same plant at a dose of 100 mg/kg in mice for 7 days did not modify the activities of AST and ALP, triglycerides, body weight, and anthropometric parameters. Quercetin was effective in reversing the decrease in weight and the imbalances in hepatic transaminases, urea, and creatinine in rats subjected to oxidative stress by cyclophosphamide (150 mg/kg) [40], which leads us to suggest that the response pattern of LP and CPA significantly depends on the experimental model used, the form of extract administration, the dose, and the treatment time.

Cyclophosphamide is oxidized by P450 enzymes in the liver to become pharmacologically active, where it is converted to highly toxic metabolites—acrolein and phosphoramide mustard [45]—which induce oxidative stress and mutagenesis. 

Regarding the micronucleus test, CPA induced a significant increase in PCEMN compared to that in the control, as observed in other studies employing mouse bone marrow cells [46,47,48]. CPA causes chromosomal damage by covalently binding to DNA and interfering with the cell cycle [49]. The liposome containing the *P. heptaphyllum* extract showed antimutagenic activity in the LP + CPA group. Furthermore, LP per se was not mutagenic. The chemoprotective effect attributed to medicinal plants is largely due to the bioactive compounds present, such as flavonoids [50].

Recent studies report similar results with extracts of plants rich in flavonoids. For example, nanoparticles from *Rhaphidophora pinnata* (50, 100, and 200 mg/kg) demonstrated antimutagenic activity against cyclophosphamide (50 mg/kg) [51]. The methanolic extract of *Dalbergia latifolia* revealed antimutagenic potential against cyclophosphamide (100 mg/kg) [52], and the flavonoid from *Kigelia africana* demonstrated antimutagenic activity against oxidative stress induced by cyclophosphamide (100 mg/kg) [53]. Considering the anti-inflammatory activity of other compounds present in *P. heptaphyllum*, the essential oil present in the resin of this plant showed, through studies of its chemical composition, cytotoxic action in breast cancer cells (MCF-7), antimicrobial activity, and antimutagenicity in vivo [54]. The possible chemopreventive activity of *P. heptaphyllum* resin essential oil is attributed to monoterpenes, in addition to the absence of cytotoxic and pro-apoptotic effects. Thus, the antioxidant activity of *P. heptaphyllum* [55,56] could potentially explain the antimutagenic activity observed in the present study, since the generation of reactive oxygen species (ROS) and oxidative stress plays a critical role in DNA and chromosome damage [57,58,59].

Animal research conducted in recent decades has demonstrated that CPA-induced hepatotoxicity is related to oxidative stress [39,43], as it triggers many liver deficiencies due to the generation of ROS [60,61]. The administration of CPA into liver tissue resulted in increased protein carbonylation in the CPA group, which is consistent with the metabolite acrolein that is incorporated into the proteins, generating carbonyl derivatives [62]. On the other hand, there was a decrease in CAT activity in the LP + CPA group compared to that in group C, an increase in GST in the LP + CPA group compared to that in the LP group, and a decrease in GSH in all treated groups (CPA, LP, and LP + CPA), and although not significant, there was a trend towards an increase in GPx for all treatments. In this sense, the GSH depletion caused by CPA is due to the production of the metabolite acrolein, which is able to form conjugations with GSH, reducing its cellular level [63]; however, GSH plays an important role in protecting cells against oxidative damage [64]. Furthermore, studies carried out by Kaushik and Kaur [65] showed the modulation of GST and GPx enzymes in relation to their coenzyme GSH during cold-induced oxidative stress for 21 days and found that enzyme activity increased, even in the presence of low GSH. It is also noteworthy that CPA demonstrated a tendency to increase CAT activity in this organ, and LP seemed to normalize this action, possibly due to the presence of bioactive compounds in the liposomes. Research using EAF from *P. heptaphyllum* in mice also showed the normalization of enzyme activity and GSH levels after paracetamol-induced oxidative stress [14]. Furthermore, a study using triterpenes from *P. heptaphyllum* restored hepatic GSH levels depleted by paracetamol in mice [5]. 

It is known that GST is involved in cellular detoxification, and its increase in the LP + CPA group may be related to an adaptive response of the liver to the stress caused by CPA. A study with *Carica papaya* Linn extract triggered an increase in GST and CAT activity in the livers of mice exposed to a single dose of CPA (75 mg/kg) [46]. Although there were changes in the redox state of the liver, no changes were observed in the immunological and histological parameters in the tissues of the groups analyzed. Similar to the results observed in this study, Patias et al. [18] did not see changes per se in LP for histological analyses and for TNF-α, IL-6, IL-17, but observed a positive effect for IL-10, and in obese animals, for IL-1β, which suggests that the animal species interferes with the cytokine response.

In renal tissue, markers of lipid and protein damage were not modified by the treatments, except in the case of LP, which showed a physiological reduction in TBARS. Furthermore, it revealed a significant increase in GPx activity in the LP + CPA group and a reduction in GST for all treatments. Contrary to our findings, studies with *Solanum scabrum* and *Cola verticillata* extracts, plants rich in flavonoids, demonstrated a decrease in renal GPx activity in rats exposed to CPA (100 mg/kg) [66]. It appears that the *P. heptaphyllum* liposome induced a response that requires greater GPx activity, possibly signaled by an additional production of hydrogen peroxide and also due to the reduction in GST. In the work of Patias et al. [14], EAF did not promote changes in GST activity per se, such as those noted in this study. On the other hand, in this study, the form of delivery of the extract in the liposomes in the group treated only with extract resulted in a decrease in TBARS. Although we observed changes in some biomarkers, the mutagenesis inducer was not effective in causing damage within 24 h of administration in the renal tissue, and consequently, the LP did not cause the activation of the immune response or the immunomodulation of the cytokines.

In brain tissue, we observed an increase per se in SOD enzyme activity in the LP group, and its activity returned to control levels in the presence of CPA compared to the levels in the LP group, which suggests that the presence of CPA may interfere with the activity of this enzyme in this tissue. The brain is a tissue composed mostly of lipids; thus, it is highly vulnerable to oxidation, and it is noteworthy that CPA did not induce changes to the point of causing damage in this tissue, which can be observed by the absence of statistical differences between the tissues of the groups studied. There are reports that curcumin improves redox balance and shows protection against oxidative damage induced by cyclophosphamide (150 mg/kg) in the brains of rats [67], and quercetin showed promising neuroprotective effects against brain oxidative damage induced by cyclophosphamide in several studies [42,68]. Furthermore, flavonoids such as quercetin, apigenin, and genistein have demonstrated the ability to reverse dysfunction caused by oxidative stress in brain endothelial cells [69]. These findings collectively highlight the neuroprotective potential of flavonoids in combating CPA-induced brain stress. In addition, we can suggest that the short time (24 h) of CPA treatment in this study was insufficient to generate brain damage, but the 14-day treatment with LP was sufficient to positively modulate SOD enzyme activity.

In cardiac tissue, there was a tendency for reduced SOD activity in the CPA group, but pretreatment with LP promoted a significant increase in this activity, suggesting that the liposome promoted an improvement in enzymatic activity. On the other hand, there was a statistical decrease in ASA levels in the CPA group, indicating a general reduction in the antioxidant capacity of this marker in cardiac tissue due to treatment with CPA, and the *P. heptaphyllum* liposome was unable to modify this parameter. Unlike the results of our investigation, the work of Ye et al. [70], in which animals were orally treated with the flavonoid chrysin (25 and 50 mg/kg/day) for 35 days and exposed to cyclophosphamide (100 mg/kg) once a week for four weeks, showed that the activities of cardiac antioxidant enzymes, such as SOD and CAT, as well as the GSH levels, were suppressed. Studies by Luiz et al. [45] also did not observe major changes in oxidative stress parameters in the hearts of mice treated with CPA (75 mg/kg) for 24 h, as was the case in our study. It is likely that this short time of exposure to CPA was not enough to cause many changes in the parameters investigated in this study for the brain and heart, but considering the results for the liver, which is the organ that metabolizes this drug, and the kidney, which is the site of excretion, these tissues did demonstrate that they are more affected by the treatment, as observed in our findings.

## 5. Final Considerations

The data show that *P. heptaphyllum* liposomes may have an antimutagenic effect, suggesting that they may act as a protective agent against DNA damage caused by CPA, including providing protection against oxidative stress, as evidenced by increased activities of the antioxidant enzymes SOD in the brain and heart, and by the increase in GPx in the kidneys. However, the depletion of GSH in the liver and brain suggests the use of antioxidants for the metabolism of molecules generated in these tissues. The improvements in ALP and creatinine markers indicate a possible hepatoprotective and renoprotective effect. Overall, these results highlight the need for further studies to fully elucidate the mechanisms of action of *P. heptaphyllum* liposomes.

## Figures and Tables

**Table 1:** Body and organ weights and food and water intake of male Swiss mice treated with LP (protium liposome) and CPA (cyclophosphamide).

Parameters	Control (C)	CPA	LP	LP + CPA
Initial body weight (g)	41.75 ± 5.23	41.12 ± 2.74	41.37 ± 2.92	39.75 ± 3.73
Final body weight (g)	44.12 ± 5.54	42.50 ± 2.13	41.12 ± 2.90	42.37 ± 3.70
Feed consumption (g/day/mouse)	44.33 ± 9.64	47.00 ± 14.53	45.00 ± 10.23	47.83 ± 13.12
Water intake (mL/day/mouse)	28.73 ± 4.21	41.03 ± 13.04	31.20 ± 6.83	29.46 ± 6.89
Liver (g)	1.72 ± 0.13	2.06 ± 0.11 *	1.81 ± 0.12	1.63 ± 0.11 **^,#^
Kidney (g)	0.51 ± 0.05	0.56 ± 0.05	0.56 ± 0.05	0.51 ± 0.03
Heart (g)	0.19 ± 0.008	0.22 ± 0.01 *	0.21 ± 0.02	0.19 ± 0.02 ^#^
Brain (g)	0.43 ± 0.05	0.42 ± 0.04	0.43 ± 0.06	0.43 ± 0.08

Results are presented as mean ± standard deviation using ANOVA (two-way), followed by Tukey’s post-hoc test. * *p* < 0.05 vs. C; ** *p* < 0.05 vs. LP; # *p* < 0.05 vs. CPA; (n = 8).

**Table 2:** Analysis of plasma parameters of male Swiss mice treated with LP (protium liposome) and CPA (cyclophosphamide).

Parameters	Control (C)	CPA	LP	LP + CPA
Glucose (mg/dL)	229.25 ± 57.17	224.62 ± 44.51	185.12 ± 43.79	251.75 ± 53.54
AST (U/L)	154.87 ± 42.00	185.50 ± 150.00	182.25 ± 97.00	186.12 ± 181.00
ALP (U/L)	112.62 ± 25.39	100.12 ± 19.65	96.62 ± 18.57	76.87 ± 7.84 *
Creatinine (mg/dL)	3.18 ± 0.66	2.13 ± 1.03 *	1.31 ± 0.46 *	1.45 ± 0.50 *
Cholesterol (mg/dL)	85.87 ± 14.24	79.80 ± 17.72	102.88 ± 32.83	95.30 ± 25.04
Triglycerides (mg/dL)	145.25 ± 16.62	126.00 ± 3.84 *	128.12 ± 16.27	136.12 ± 5.41

Results are presented as mean ± standard deviation using ANOVA (two-way), followed by Tukey’s post-hoc test. For AST analysis, a Kruskal–Wallis test, followed by Dunn’s post-hoc test, was used, and values were expressed as median and total range. * *p* < 0.05 vs. C. (AST: aspartate aminotransferase; ALP: alkaline phosphatase); (n = 8).

**Table 3:** Frequency of micronucleated polychromatic erythrocytes (PCEMN) in bone marrow of male Swiss mice treated with LP (protium liposome) and CPA (cyclophosphamide).

	PCEMNs			
Treatment	Number of PCEs Analyzed	MN	%	% Reduction
Control (Water + NaCl 0.9%)	8000	290	3.62	
CPA (Water + CPA)	8000	535	6.69	
LP (LP + NaCl 0.9%)	8000	264	3.30	
LP + CPA (LP + CPA)	8000	470 *	5.81	26%

* *p* < 0.05 in comparison with the CPA group, according to the Chi-squared test; (n = 8).

**Table 4:** Analysis of cytokines in liver and kidney tissue of male Swiss mice treated with LP (protium liposome) and CPA (cyclophosphamide).

	Parameters	Control (C)	CPA	LP	LP + CPA
Liver	TNF-α (pg/mL)	0.72 ± 0.26	0.76 ± 0.24	0.83 ± 0.25	0.97 ± 0.15
	IFN-γ (pg/mL)	1.17 ± 0.19	1.20 ± 0.12	1.24 ± 0.11	1.27 ± 0.07
	IL-6 (pg/mL)	1.05 ± 0.18	1.12 ± 0.14	1.13 ± 0.10	1.15 ± 0.04
	IL-10 (pg/mL)	1.15 ± 0.26	1.27 ± 0.17	1.28 ± 0.10	1.37 ± 0.08
	IL-17 (pg/mL)	0.97 ± 0.22	1.11 ± 0.24	1.13 ± 0.17	1.18 ± 0.11
	IL-β (pg/mL)	0.77 ± 0.22	0.89 ± 0.11	0.97 ± 0.17	1.01 ± 0.13
Kidney	TNF-α (pg/mL)	0.96 ± 0.21	1.15 ± 0.32	0.93 ± 0.30	0.94 ± 0.21
	IFN-γ (pg/mL)	1.30 ± 0.14	1.46 ± 0.09	1.34 ± 0.20	1.42 ± 0.19
	IL-6 (pg/mL)	1.21 ± 0.18	1.38 ± 0.20	1.28 ± 0.25	1.24 ± 0.23
	IL-10 (pg/mL)	1.24 ± 0.18	1.39 ± 0.11	1.31 ± 0.27	1.28 ± 0.27
	IL-17 (pg/mL)	1.25 ± 0.13	1.16 ± 0.17	1.09 ± 0.23	1.17 ± 0.13
	IL-β (pg/mL)	1.00 ± 0.23	1.16 ± 0.36	1.23 ± 0.28	1.12 ± 0.26

Results are presented as mean ± standard deviation. ANOVA (two-way) followed by Tukey’s post-hoc test; (n = 8). For statistical analysis, data were transformed into sqrt.

**Table 5:** Analysis of redox status parameters in the liver and kidney tissue of male Swiss mice treated with LP (protium liposome) and CPA (cyclophosphamide).

	Parameters	Control (C)	CPA	LP	LP + CPA
Liver	GSH (µmol de GSH/mg protein)	3.65 ± 1.15	1.96 ± 0.49 *	2.60 ± 0.53 *	2.36 ± 0.64 *
	ASA (μmol ASA/g tissue)	3.05 ± 0.48	3.33 ± 1.04	2.73 ± 0.49	3.28 ± 0.97
	TBARS (nmol MDA/mg protein)	0.71 ± 0.20	0.89 ± 0.19	0.56 ± 0.18	0.66 ± 0.42
	Carbonyl (nmol carbonyl/mg protein)	13.60 ± 2.98	19.06 ± 3.76 *	15.30 ± 4.73	17.16 ± 3.55
Kidney	GSH (µmol de GSH/mg protein)	2.82 ± 3.65	3.53 ± 5.54	1.68 ± 4.08	2.35 ± 5.68
	ASA (μmol ASA/g tissue)	1.40 ± 0.22	1.68 ± 0.21	1.38 ± 0.35	1.42 ± 0.33
	TBARS (nmol MDA/mg protein)	0.10 ± 0.15	0.09 ± 0.05	0.07 ± 0.05 *	0.08 ± 0.07
	Carbonyl (nmol carbonyl/mg protein)	5.35 ± 2.25	5.99 ± 1.41	6.08 ± 2.02	4.89 ± 2.27

The results are presented as mean ± standard deviation. ANOVA (two-way), followed by Tukey’s post-hoc test. For analysis of GSH and TBARS of the kidney, the Kruskal–Wallis test, followed by Dunn’s post-hoc test, was used, and the values were expressed as median and total range. * *p* < 0.05 vs. C. (GSH: reduced glutathione; ASA: ascorbic acid; TBARS: thiobarbituric acid reactive substances; Carbonyl: carbonylated proteins); (n = 8).

**Table 6:** Analysis of redox status parameters in brain and heart tissue of male Swiss mice treated with LP (protium liposome) and CPA (cyclophosphamide).

	Parameters	Control (C)	CPA	LP	LP + CPA
Brain	GSH (µmol de GSH/mg protein)	2.60 ± 3.42	3.67 ± 3.35	1.65 ± 1.30	1.93 ± 1.90 ^#^
	ASA (μmol ASA/g tissue)	3.46 ± 0.34	3.31 ± 0.32	3.54 ± 0.40	3.13 ± 0.26
	TBARS (nmol MDA/mg protein)	4.57 ± 1.40	5.15 ± 0.72	4.41 ± 0.66	5.00 ± 0.87
	Carbonyl (nmol carbonyl mg protein)	18.45 ± 4.25	17.37 ± 3.40	24.30 ± 6.87	23.50 ± 3.08
Heart	GSH (µmol de GSH/mg protein)	1.05 ± 0.34	0.91 ± 0.32	1.81 ± 0.65	1.38 ± 0.79
	ASA (μmol ASA/g tissue)	1.77 ± 0.39	1.30 ± 0.31 *	1.52 ± 0.35	1.46 ± 0.28
	TBARS (nmol MDA/mg protein)	0.67 ± 0.21	0.63 ± 0.10	0.55 ± 0.18	0.78 ± 0.13
	Carbonyl (nmol carbonyl/mg protein)	23.56 ± 11.48	21.79 ± 18.93	25.81 ± 16.33	36.15 ± 31.28

The results are presented as mean ± standard deviation. ANOVA (two-way), followed by Tukey’s post-hoc test. For analysis of GSH of brain tissue and carbonyl of cardiac tissue, a Kruskal–Wallis test, followed by a post-hoc Dunn test, was used, and values were expressed as median and total range. * *p* < 0.05 vs. C; # *p* < 0.05 vs. CPA. (GSH: reduced glutathione; ASA: ascorbic acid; TBARS: thiobarbituric acid reactive substances; Carbonyl: carbonylated proteins); (n = 8).

## Data Availability

The data that support the findings of this study are available from the corresponding author upon reasonable request.
